# Aquatic Functional Liquid Crystals: Design, Functionalization, and Molecular Simulation

**DOI:** 10.1002/advs.202306529

**Published:** 2023-12-21

**Authors:** Takashi Kato, Junya Uchida, Yoshiki Ishii, Go Watanabe

**Affiliations:** ^1^ Department of Chemistry and Biotechnology School of Engineering The University of Tokyo Hongo Bunkyo‐ku Tokyo 113‐8656 Japan; ^2^ Research Initiative for Supra‐Materials Shinshu University Nagano 380‐8553 Japan; ^3^ Department of Data Science School of Frontier Engineering Kitasato University Sagamihara 252‐0373 Japan; ^4^ Kanagawa Institute of Industrial Science and Technology (KISTEC) Ebina 243‐0435 Japan

**Keywords:** interfaces, liquid crystals, molecular dynamics, molecular simulation, self‐organization, water

## Abstract

Aquatic functional liquid crystals, which are ordered molecular assemblies that work in water environment, are described in this review. Aquatic functional liquid crystals are liquid‐crystalline (LC) materials interacting water molecules or aquatic environment. They include aquatic lyotropic liquid crystals and LC based materials that have aquatic interfaces, for example, nanoporous water treatment membranes that are solids preserving LC order. They can remove ions and viruses with nano‐ and subnano‐porous structures. Columnar, smectic, bicontinuous LC structures are used for fabrication of these 1D, 2D, 3D materials. Design and functionalization of aquatic LC sensors based on aqueous/LC interfaces are also described. The ordering transitions of liquid crystals induced by molecular recognition at the aqueous interfaces provide distinct optical responses. Molecular orientation and dynamic behavior of these aquatic functional LC materials are studied by molecular dynamics simulations. The molecular interactions of LC materials and water are key of these investigations. New insights into aquatic functional LC materials contribute to the fields of environment, healthcare, and biotechnology.

## Graphical abstract

Aquatic functional liquid crystals, which are ordered molecular assemblies that work in water environment, have great potential for contribution to the fields of environment, resources, healthcare, and bio‐applications. In this perspective, recent progress in water treatment liquid‐crystalline (LC) polymer membranes and aquatic LC sensors is described, from design, assembly, and functionalization to molecular simulation of these aquatic functional liquid crystals.

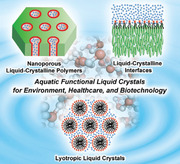

## 1 Introduction

Molecular nanoscale assemblies have attracted attention due to their dynamic and stimuli responsive properties.^[^ ^1^ ^]^ Liquid crystal, which is one of molecular assemblies has been widely studied as functional materials in a variety of fields in information, separation, energy, environment, and bio‐function.^[2−9]^ In the textbook of liquid crystals, liquid crystals are categorized into thermotropic liquid crystals and lyotropic liquid crystals. Thermotropic liquid crystals of low‐molecular‐weight compounds have been applied to informational displays. Thermotropic liquid‐crystalline (LC) main‐chain polymers are solid‐state super‐engineering plastics keeping LC order in the melt‐processing state. In contrast, lyotropic liquid crystals are related to biological systems and industrial compounds of food, cosmetics, and soaps.

**Figure 1:**
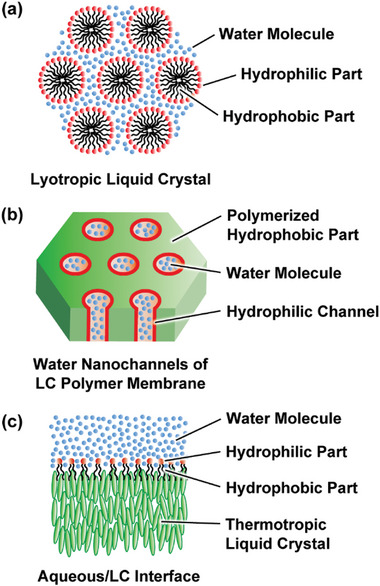
Examples of aquatic functional liquid crystals. a) Lyotropic liquid crystals composed of amphiphilic molecules and water, b) water nanochannels of LC polymer membranes, and c) aqueous/LC interfaces decorated with amphiphilic molecules.

The most conventional and normal relationships between water and liquid crystals are lyotropic liquid crystallinity (**Figure** **1**a).^[^ ^2^, ^4^ ^]^ Lyotropic LC system forms one thermodynamically stable phase that includes water molecules as essential components. Typical and well‐recognized lyotropic LC molecules have two immiscible components consisting of amphiphilic ionic group and hydrophobic hydrocarbon group. In this case, water molecules are a component of hydrophilic domain of nano‐segregated liquid crystal structures.

Polymeric systems that preserve thermotropic and lyotropic LC structures contacting water molecules at their interface of nano‐ or subnano‐porous channels are one of aquatic functional liquid crystals^[^ ^8^ ^]^ (Figure 1b). For these new aquatic functional materials, nanostructured LC materials have been shown to be useful for water‐treatment membranes that exhibit specific properties due to ordered structures based on LC nature.^[^ ^8^ ^]^ Nanostructured LC phases,^[^ ^10^ ^]^ bicontinuous cubic, smectic, and columnar mesophases have been used to form water nanochannels. The basic strategy to prepare LC water‐treatment membranes is fixation of in situ polymerization of nanostructured liquid crystals forming nano‐channels.

Aquatic LC sensors have emerged in these two decades, which extend potentials of new functions of molecular assemblies (Figure 1c).^[^ ^9^ ^]^ These LC sensors provide distinct changes in optical appearance under crossed polarizers in response to target chemical and biological species in water. The specific binding events at aqueous/LC interfaces cause ordering transitions of thermotropic liquid crystals, which is amplified to generate macroscopic optical signals. These stimuli‐responsive aqueous/LC interfaces have been applied to LC thin films^[^ ^9a,b^ ^]^ and LC droplets in water.^[^ ^9c^ ^]^

To understand the molecular behavior and properties of these aquatic functional liquid crystals, molecular simulation is expected to be a powerful tool. For over 30 years, the structures of a variety of liquid crystals have been studied by means of molecular simulations, especially molecular dynamics (MD) simulations.^[^ ^11^ ^]^ At the early stage, simple hard particle models have been used for the molecular simulations to understand the physical mechanism of liquid crystals.^[^ ^12^ ^]^ With the growth of computer performance, the role of the molecular simulations has been changed: not only investigating the effect of the molecular shape on the assembly formation but also understanding the chemical insights of dynamics, structures, and interactions of liquid crystals at the atomic level. Since early 2000s,^[^ ^13^ ^]^ united atom models in which CH_3_ and CH_2_ alkyl groups have been treated as one unified interaction site are widely used to save the computation cost. The nematic‐isotropic phase transitions of 4,4′‐alkyl‐cyanobiphenyls (*n*CB) and E7, a mixture of four their related compounds have been well reproduced.^[^ ^13^, ^14^, ^15^ ^]^ Moreover, the columnar mesophase, cubic phases, and monolayers at the water interface have been well demonstrated.^[^ ^16^ ^]^ However, any investigation of the sophisticated LC structures had been still difficult with all‐atom models because of the issue of machine power at the time. In the recent decade, on the benefit of the steady increase of the computer power, more realistic all‐atom MD simulations of the LC systems have come within reach. Thereby, in the present era, molecular simulations of LC molecules meet a next challenge of aquatic functional materials bringing new structural and dynamic features. It is also important to prepare the large‐scale system for avoiding finite‐size effects and run the sufficient long time for equilibration.

As a modern LC simulation, lyotropic LC systems and their related aquatic molecular assemblies consisting of both organic and water molecules have been studied by MD simulations. All‐atom MD simulations performed for micelles, lamellas, and H‐ and J‐aggregate sheet structures in aqueous solution, and the structure and dynamics of LC phases have been well discussed.^[^ ^17^ ^]^ The effects of some cations on the dynamics of water confined in these lyotropic LC morphologies have been investigated in the MD simulation study of gyroid and hexagonally‐packed cylinders formed by anionic Gemini surfactants molecules with counterions.^[^ ^18^ ^]^

Compared to bulk LC systems, MD simulations have not been widely used to study monolayers and thin films at the interface. For developing practical LC materials, the interaction of LC molecules with the interface, such as anchoring, is the important physical properties. Since it is still challenging to observe the molecular alignment near the surface by the experiments, several MD simulation studies were reported for investigating the behavior of the LC molecules at solid interfaces.^[^ ^19^ ^]^

Due to the difficulty in obtaining the relevant nanoscale‐level detailed information regarding to interfacial structure at aqueous/LC interfaces using the experimental tools, the monolayer and thin films at the water interface have been considered to be difficult systems for accurate studies by MD simulations. During these several years, the large‐scale atomistic MD simulations on LC phases of typical mesogenic molecules in the vicinity of vacuum and aqueous interfaces have been performed.^[^ ^20^ ^]^ While understanding the microscopic dynamics is a key factor to design the aquatic functional LC materials, only a few MD simulation studies have been reported to address the dynamic and structural properties.^[^ ^16^, ^20^, ^21^ ^]^

**Figure 2:**
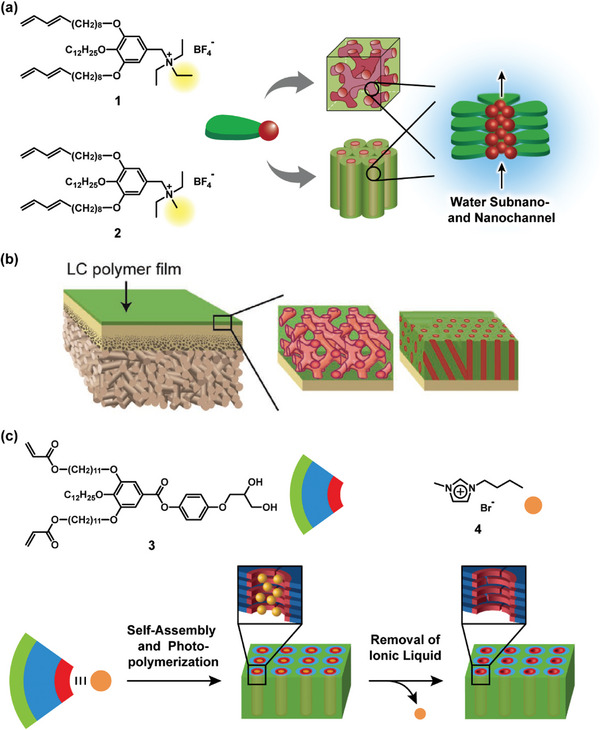
a) Chemical structures of thermotropic LC molecules **1** and **2** exhibiting bicontinuous and columnar LC phases, respectively. These molecules form water subnano‐ and nanochannels in the LC phases. b) Schematic illustration of the nanostructured LC composite membranes. c) Chemical structures of fan‐shaped molecule **3** and ionic liquid **4**. Larger nanopores can be obtained by photopolymerization of the columnar LC mixtures of **3** and **4** and subsequent removal of **4** from the membranes. b) Reproduced under the terms of the CC‐BY Creative Commons Attribution 4.0 International license (https://creativecommons.org/licenses/by/4.0).^[^ ^8f^ ^]^ Copyright 2018, The Authors, published by Wiley‐VCH. c) Adapted with permission.^[^ ^23h^ ^]^ Copyright 2019, American Chemical Society.

In this perspective, we focus on some of new aspects of water‐based liquid crystals including design, preparation, functionalization of LC molecular assemblies. We consider functionalization of liquid crystals in aquatic environment is important for contribution of the materials to the field of environment, resources, healthcare, and bio‐applications. One of representative liquid crystals in aquatic environment is lyotropic liquid crystal such as lipids (Figure 1a). They form one LC phase. In this perspective we describe aquatic LC materials that show functions consisting of two phases of LC structures and aqueous phases: 1) subnano‐ and nano‐porous water treatment membranes consisting of fixed LC structures and porous aquatic channels consisting of water molecules (Figure 1b); 2) aquatic LC sensors consisting of room temperature liquid crystals and aquatic phases consisting of water molecules (Figure 1c). For these phase‐separated materials, the interactions of LC materials at the interfaces and water molecules play critical roles. These new viewpoints may generate new functions and directions of liquid crystals. We also show studies of molecular simulation on these aquatic liquid crystals as examples of collaboration of experiment and computer science for design and understanding of these molecular assemblies.^[^ ^6^, ^21^ ^]^

## 2 Self‐Organized Liquid‐Crystalline Water Treatment Membranes

Water treatment by reverse osmosis (RO) and nanofiltration (NF) membranes has been used widely as technology to obtain pure and clean water.^[^ ^8^, ^22^ ^]^ These RO and NF membranes consisting cross‐linked aromatic polyamides have subnano‐ and nano‐scale water channels, which have size distributions. Water‐treatment solid‐state membranes keeping the LC nanostructures with regular pore sizes have been developed.^[^ ^8^, ^23^ ^]^ They are considered to be aquatic functional liquid crystals. Water molecules permeate through the nano‐channels of the membranes formed by in‐situ polymerization of low‐molecular‐weight monomers in the nano‐segregated LC phases. For these materials, 1D, 2D, and 3D nanochannels were obtained by self‐assembly of columnar, smectic, and bicontinuous thermotropic liquid crystals.^[^ ^24^ ^]^ One of representative monomers that form 3D and 1D nanochannel structures are shown in **Figure** **2**a. It is of interest that compound **1**, a taper shaped molecule with triethylammonium moiety exhibited a bicontinuous cubic phase,^[^ ^8^, ^25^ ^]^ while compound **2** having a diethylmethylammonium moiety showed a columnar phase.^[^ ^8f^ ^]^ Basically, we understand the formation of these different LC phases by a geometric model based on the radial distribution of volume for the taper shaped molecules.^[^ ^26^ ^]^ The volume of the ionic moieties at the focal point for **1** is larger than that for **2**, which is considered to affect the self‐assembled structures in the LC phases due to the change of balance between ionophobic and ionophilic moieties. However, the formation of the bicontinuous cubic phases is not easy to design because it is induced by the very delicate balance.^[^ ^27^ ^]^ This is still an issue in the design of nanostructured LC materials. It is noteworthy that water nanochannels were formed in these thermotropic LC phases obtained without water molecules (Figure 2a,b). The pore size of the LC membrane prepared from **1** was estimated to be 0.6 nm by positron‐annihilation lifetime spectroscopy (PALS).^[^ ^8e^ ^]^ The size of pores for the membrane based on **2** was assumed to be similar to that for **1** because water flux of **2** was almost the same as that of **1**.^[^ ^8f^ ^]^ Moreover, the pore sizes of the LC membranes were tuned by using two‐component columnar liquid crystals (Figure 2c).^[^ ^23h^ ^]^ Photopolymerization of the columnar LC mixtures of fan‐shaped molecule **3** and ionic liquid **4** provided nanostructured polymer membranes. Subsequent removal of the ionic liquid from the membranes led to the formation of larger nanopores.^[^ ^23h^ ^]^

The functions of the LC polymer membranes prepared from **1** and **2** were expected to be different from those of conventional RO membranes due to the presence of cationic moieties on the surface of the nanochannels. Ion permeation experiments showed that these membranes were capable of permeating ions selectively.^[^ ^8e,f^ ^]^ The permeation of divalent sulfate ions was higher than that of monovalent chloride ions even though the sulfate ions were larger than chloride ions. This behavior was explained by structural consistency of hydrogen‐bonded structures of water molecules inside the ionic pores and those surrounding the hydrated ions.^[^ ^28^ ^]^ The energy states of water molecules inside the ionic nanochannels were also studied by MD simulation,^[^ ^21c^ ^]^ which are described below. The insights obtained by the combination of advanced measurements and simulations of these water treatment LC membranes may be useful for the further design of ion‐selective nanochannels.^[^ ^29^ ^]^

**Figure 3:**
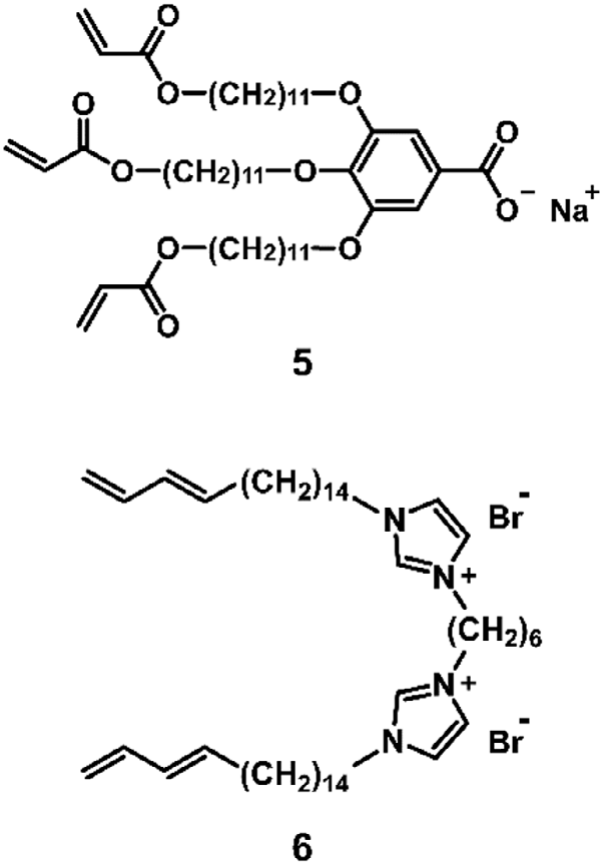
Chemical structures of compounds **5** and **6** forming lyotropic LC phases.

Lyotropic LC‐based nanostructured polymer membranes have been developed by Gin, Noble, and coworkers.^[^ ^8^, ^30^, ^31^, ^32^, ^33^ ^]^ For example, polymerizable wedge‐shaped compound **5** having a carboxylate group was synthesized and used as a monomer for the formation of water‐treatment LC membranes (**Figure** **3**).^[^ ^8^, ^31^ ^]^ Compound **5** exhibited inverted hexagonal lyotropic LC phases in the presence of water, where the diameter of the water nanochannels was estimated to be ≈1 nm. The hexagonally ordered LC nanochannels were crosslinked by photopolymerization of the lyotropic LC assemblies. These LC membranes supported on porous polymer substrates showed size‐selective filtration of molecules in water.^[^ ^31^ ^]^ Moreover, macroscopic vertical alignment of the hexagonally packed nanopores formed by self‐assembly of **5** was demonstrated by applying magnetic fields^[^ ^32a^ ^]^ and soft confinement.^[^ ^32b^ ^]^ In addition to the hexagonal LC assemblies, bicontinuous cubic lyotropic LC assemblies were employed for the development of water‐treatment LC membranes.^[^ ^30^, ^33^ ^]^ Imidazolium‐based gemini monomer **6** (Figure 3) was mixed with glycerol instead of water to form a bicontinuous cubic lyotropic LC phase.^[^ ^30^, ^33^ ^]^ They use two‐component strategy.^[^ ^23^, ^34^ ^]^ The use of glycerol as an additive liquid components with low volatility allowed the formation of thin‐film composite membranes by solution‐based processes due to the minimal evaporation of glycerol and retention of the bicontinuous lyotropic LC phase. The bicontinuous LC composite membranes exhibited rejection of molecular solutes based on size exclusion and high salt rejection. Similar approach using nonvolatile components for the development of thin LC membranes was applied to lyotropic LC materials forming normal‐type hexagonal phases.^[^ ^35^ ^]^ In addition, nanoporous LC polymer materials have been used as selective adsorbents.^[^ ^23^, ^36^ ^]^

**Figure 4:**
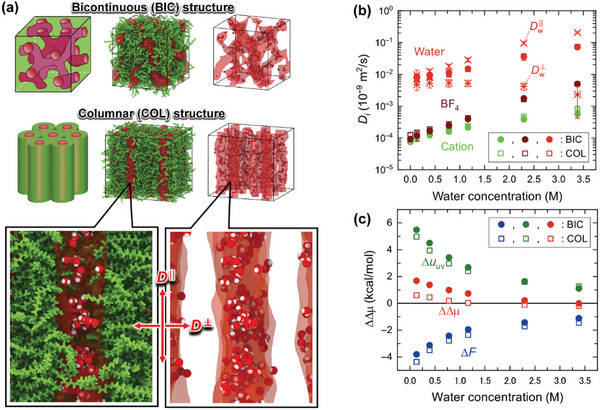
a) Self‐assembled bicontinuous and columnar nanostructures of compound **1** obtained by all‐atom MD simulation and the enlarged view of ionic nanochannels inside the columnar self‐assembly. The hydrophobic moieties of compound **1** are illustrated with green bonds, and the nanosegregated domain with the ionic moieties is depicted with transparent red pipe. The bottom left and right panels are shown with and without hydrophobic bonds, respectively, of compound **1**. b) Self‐diffusion coefficients of water and compound **1** in the self‐assembled bicontinuous (BIC) and columnar (COL) structures. For water molecules, the longitudinal and transverse diffusion coefficients, *D*_w_^||^ and *D*_w_^⊥^, are further analyzed in the self‐assembled columnar structure with the direction of ionic nanochannels shown in (a). c) Solvation free energy change ΔΔμ of water molecules between the media of water and self‐assembled LC system. The partial contributions of ΔΔμ are decomposed from the differences of solute‐solvent interaction and solvent‐reorganization effect, Δ*u*_uv_ and Δ*F*, between the solvent media of water and self‐assembled LC system. Copyright 2021, The Authors, published by American Association for the Advancement of Science. Reprinted/adapted from ref. [21c]. The Authors, some rights reserved; exclusive licensee American Association for the Advancement of Science. Distributed under a Creative Commons AttributionNonCommercial License 4.0 (CC BY‐NC) https://creativecommons.org/licenses/by‐nc/4.0/.

Molecular simulations for compound **1** have been recently achieved with the fully‐atomistic resolution as the explicit observation of self‐assembled nanostructures.^[^ ^21c^ ^]^ The utilized molecular model was newly developed within the density functional theory of electrostatic state in a condensed system.^[^ ^37^ ^]^ The atomic charges of ionic molecules were then tuned to take account of electrostatic interaction in the LC state. The refined MD simulation of compound **1** successfully provides the self‐assembled bicontinuous and columnar structures at room temperature (**Figure** **4**). Hidden nanochannels in the bicontinuous self‐assembly are interconnected as the 3D network domain (Figure 4a). The 1D columnar structures are also formed for **1** (Figure 4a). These observations show that the recent methodology of theoretical chemistry allows us to access some properties of self‐assembled LC structures with sufficient validity of molecular simulation. Meanwhile, in the real system, compound **1** forms only the bicontinuous LC phase around room temperature, and the columnar LC phase is exhibited in compound **2**. Thus, the LC phase behavior is determined by the chemical structures of ionic groups: the exact dependence of self‐assembled nanostructures for ionic LC compounds has not yet been reproduced within the framework modern molecular simulation. It would be one of the challenges of molecular simulation for materials chemistry.

**Figure 5:**
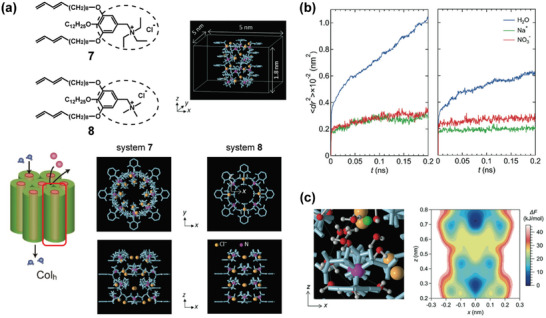
a) Chemical structures of compounds **7** and **8** and their snapshots of the subnanochannels of systems **7** and **8**, respectively, formed with the ionic moieties of LC molecules. The distances of nitrogen atoms from the pore center in the *x*–*y* plane were set to 0.67 and 0.45 nm for systems **7** and **8**, respectively. The illustration of self‐assembled columnar LC structure is also depicted in the left panel, and the red square show the ideal single column of self‐assembled LC phase. b) Mean square displacements (MSDs) of water, Na^+^, and NO_3_^−^ obtained by MD simulation for the NaNO_3_ aqueous solutions inside the subnanohannels of systems **7** and **8**. The left and right panels of MSDs were observed for compounds **7** and **8**, respectively. c) Free‐energy landscape projected onto the *x*–*z* plane for Na^+^ ion in the NaCl aqueous solution in the subnanochannel for system **7**. The free‐energy difference Δ*F* was obtained from a reference position at the lowest value at (*x*, *z*) = (0.0, 0.72). In the left panel of (c), the green sphere represents Na^+^ ion, and the O and H atoms of water molecules were displayed with the red and white spheres, respectively. Adapted with permission.^[^ ^21a^ ^]^ Copyright 2020, Royal Society of Chemistry.

The self‐assembled LC monomers of **1** and **2** were photopolymerized in the self‐assembled LC states on polymer substrates, resulting in the formation of nanoporous polymer composite membranes. These membranes were used for water treatment. The water molecules can permeate the membranes through the nanochannels. The cross‐linking and cross‐liked structures have not yet been studied by simulation. However, we were able to examine the interactions of water molecules inside the nanochannels of self‐assembled LC structures by some MD studies.^[^ ^21^, ^38^ ^]^ Nada and coworkers investigated dynamics of confined water and ions inside the nanochannels of compounds **7** and **8** in response to nanopore size through an MD simulation of the single column condition (**Figure** **5**a).^[^ ^8^, ^21^ ^]^ In this case, some atoms of LC molecules were fixed for the nanochannel environment. Water molecules then move freely as the random walk, while the jump (cage) diffusion motion can be observed for Na^+^ and NO_3_^−^ ions (Figure 5b). The free‐energy profile supported that ions remain trapped in local sites of the nanochannel (Figure 5c). Furthermore, it should be noted that the chargeless condition of LC molecules clearly damped the mobility of water and ions inside the nanochannel.^[^ ^21a^ ^]^ Thereby, electrostatic interactions inside the nanochannels may play an important role to exhibit water and ion transport properties of self‐assembled LC membranes.

The hydration properties of nanochannels have been further analyzed with the fully atomistic resolution of self‐assembled LC phases for compound **1** through large‐scale MD studies.^[^ ^18^, ^21^, ^38^ ^]^ In the self‐assembled LC structures of compound **1**, the positions of water and ionic LC molecules were relaxed without any artificial restriction. The nanochannels of self‐assembled bicontinuous and columnar structures retained water molecules inside the hydrophilic domain. Here, this nanoconfined environment was preserved with the electrostatic interaction of ionic moieties for compound **1** and allowed to form hydrogen bonds along the direction of nanochannel networks (Figure 4a). The binding free energies between the ionic moieties of anion and cation were then comparable between the self‐assembled bicontinuous and columnar structures. In the nanoscale aqueous electrolyte solutions, the diffusivity of water molecules was enhanced with an increase to the water content. The mobility of BF_4_ anion for compound **1** also increased slightly rather than the cation (Figure 4b). The potential of mean force for water and ions then supported that the hydration of BF_4_ anions reduces the energy barrier of diffusion for anions inside the nanochannels.^[^ ^21c^ ^]^ Meanwhile, the difference of self‐assembled LC structures may remind small for isotropic properties of water transport. Here, from the potential energy profile of water molecules, microscopic states of water molecules can be identified in response to local regions of the self‐assembled nanostructure (Figure 4a). The hydrophobic domains for the outside of ionic nanochannels were relatively unfavorable for water molecules. The transverse diffusion across the nanochannels was also slightly observed (Figure 4a,b), so that the energy barrier of hydrophobic self‐assembly domain among the nanochannels was found to be large for water molecules.^[^ ^21c^ ^]^ Inside the nanochannel of self‐assembly, confined water molecules interacted with other water molecules and the ionic moieties of cation and anion. The structural preference of confined states changes in response to the water content. In comparison of the solvation free energies between water and self‐assembled LC structures, the free energy change beyond the interface of aqueous solution and LC membrane phases became smaller with an increase of the water content inside the nanochannel (Figure 4c). Then, the intermolecular interactions with surrounding molecules remained more favorable in aqueous solution than with nanochannels in the self‐assembled LC membrane. However, in the nanochannel medium, the energy‐loss advantage of cavity formation profited for the process of water transport (Figure 4c). After water transport into the nanochannel environment, the formation of hydrogen bonding network enhanced the stability of confined water molecules in the ionic nanochannel. As a result, only the longitudinal diffusion of water along the nanochannel direction was emphasized in response to the water content (Figure 4a,b). Here, we discuss structural effect of self‐assembled LC phases again. The formation of ionic nanochannels of the bicontinuous and columnar LC phases is delicate. After the photopolymerization, water and ions are filtered through the nanochannel environment. Therefore, the diffusion and energetic process of water transport onto the direction of ionic nanochannels is of key importance for the water treatment membrane. Here, the ordering structure of ionic moieties in the columnar self‐assembly advantages to a reduction of solvation free energy of confined water.^[^ ^21c^ ^]^ The self‐assembled LC geometry of 1D nanochannel slightly profits the nature of water transport via advantages of energetic and diffusion mechanisms inside the nanochannel environment under the dry and hydrated conditions, respectively.^[^ ^21c^ ^]^ Water‐transport behavior through the ionic nanochannels was also improved in the case of 1D aligned nanodomain. These advantages were thereby provided with mesostructure and its nanoscale refinement of the ionic nanochannels in the self‐assembled LC membranes. Shirts and coworkers also investigated the transport properties of water and neutral polar molecules for the lyotropic LC membranes of compound **5** designed by Gin and coworkers.^[^ ^38^ ^]^ They computed the diffusivity of confined molecules inside the ionic nanochannels by combining MD method and advanced Markov‐state models for long‐timescale behavior.^[^ ^38c,d^ ^]^ These statistical‐mechanical analyses at atomic resolution played significant roles to elucidate microscopic properties of water and ions and further make a guideline for imaging the molecular structures of self‐assembled LC membrane.

## 3 Aqueous/Liquid‐Crystalline Interfaces for Sensing

**Figure 6:**
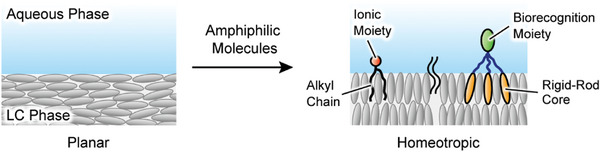
Ordering transitions of liquid crystals at the aqueous/LC interfaces induced by amphiphilic molecules.

In the last two decades, new stimuli‐responsive aquatic LC materials have been developed based on ordering transitions of thermotropic liquid crystals at the aqueous interfaces (**Figure** **6**).^[^ ^3^, ^5^, ^9^, ^39^ ^]^ In particular, the dynamic aqueous/LC interfaces have great potential for label‐free detection of target chemical and biological species in water because they exhibit distinct changes in optical appearance.^[^ ^9^ ^]^ In this section, we describe the recent progress in the design, applications, and molecular simulations of the aqueous/LC interfaces as sensing platforms.

Abbott and coworkers reported a seminal approach to tailoring interfacial properties of thermotropic liquid crystals in contact with water.^[^ ^9a–c^ ^]^ Amphiphilic molecules such as biological lipids and synthetic surfactants have been adsorbed at the aqueous/LC interfaces to control the LC alignment, responsiveness, and molecular recognition (Figure 6). The changes in LC order were attributed to the intermolecular interactions induced by interdigitation between LC molecules and hydrophobic moieties of the amphiphilic molecules (Figure 6).^[^ ^9a,b^ ^]^ These LC interfaces have been used to report intermolecular interactions with a variety of chemical and biological species on the surface of liquid crystals.^[^ ^40^ ^]^

**Figure 7:**
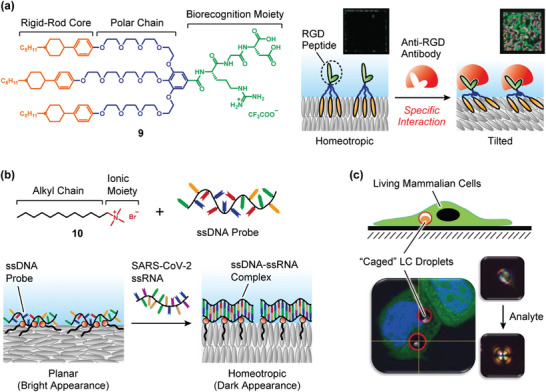
a) Chemical structures of RGD peptide‐conjugated forklike mesogen **9**. The optical appearance of LC interfaces modified with **9** was changed by specific recognition of anti‐RGD antibody. b) Detection of SARS‐CoV‐2 ssRNA at aqueous/LC interfaces modified with cationic surfactant **10** and ssDNA probe. c) “Caged” LC droplets exhibiting optical responses to toxic amphiphiles inside living cells. a) Adapted under the terms of the CC‐BY‐NC‐ND Creative Commons Attribution 4.0 International license (https://creativecommons.org/licenses/by‐nc‐nd/4.0).^[^ ^41c^ ^]^ Copyright 2023, The Authors, published by American Chemical Society. c) Adapted with permission.^[^ ^45a^ ^]^ Copyright 2021, American Chemical Society.

Functional amphiphilic mesogens bearing rigid‐rod cores have also been designed and synthesized for development of biomolecular LC interfaces (Figure 6).^[^ ^41^ ^]^ These amphiphilic mesogens mixed with nematic liquid crystals were shown to partition from the LC phases to the aqueous interfaces. These results demonstrate that the modification of the LC interfaces with functional moieties is a general and facile approach. For example, the aqueous/LC interfaces minimizing nonspecific adsorption of proteins were prepared based on interfacial partitioning of an amphiphilic mesogen having a tetraethylene glycol moiety.^[^ ^41a^ ^]^ Bioconjugated amphiphilic mesogens contained specific binding moieties such as biotin and arginine‐glycine‐aspartic acid (RGD) peptides. They were also developed to couple biorecognition functions with the aqueous/LC interfaces.^[^ ^41b–e^ ^]^ Forklike structures with multiple rigid‐rod cores^[^ ^42^ ^]^ were used to achieve distinct optical changes in response to target biomolecules. These structures can enhance the interactions between the bioconjugated mesogens and nematic liquid crystals (**Figure** **7**a).^[^ ^41c^ ^]^ RGD‐peptide conjugated forklike mesogen **9** triggered ordering transitions of liquid crystals by specific recognition of anti‐RGD antibody (Figure 7a), leading to the optical response from dark to bright appearance. Self‐assembled properties of the functionalized mesogens at the air/water interfaces as well as in the LC phases were also studied.^[^ ^41^, ^43^ ^]^ The use of designed bioconjugated mesogens may provide fundamental insights into biomolecular interactions with liquid crystals at the aqueous interfaces.

Recently, the aqueous/LC interfaces have been extensively studied for practical use as LC sensors.^[^ ^44^ ^]^ Combination of nucleic acid probes with the aqueous/LC interfaces was effective to detect disease‐related nucleic acids and proteins with high sensitivity.^[^ ^44^ ^]^ For example, a single‐stranded deoxyribonucleic acid probe (ssDNA_probe_), which can recognize the severe acute respiratory syndrome‐coronavirus‐2 (SARS‐CoV‐2) single‐stranded ribonucleic acid (ssRNA_CoV_), was adsorbed at the aqueous/LC interfaces (Figure 7b).^[^ ^44a^ ^]^ Hybridization of the ssDNA_probe_ with cationic surfactant **10** at the LC interfaces induced planar alignment of liquid crystals. In the planar LC alignment, the hydrophobic bases of the flexible ssDNA_probe_ were suggested to interact with compound **10** to decrease the effective surface coverage of **10**. Incubation of the ssRNA_CoV_ to the ssDNA_probe_‐decorated LC interfaces led to changes in optical appearance from bright to dark due to the ordering transitions of liquid crystals (Figure 7b). This orientational change of liquid crystals was ascribed to reorganization of cationic surfactant **10** triggered by the formation of rigid ssRNA‐ssDNA complexes at the LC interfaces (Figure 7b). Moreover, diagnostic kit for naked‐eye detection of ssRNA_CoV_ and smartphone‐based application were designed.

The aqueous/LC interfaces work as chemical sensors in complex cellular environments^[^ ^45a–c^ ^]^ and living organisms.^[^ ^45d^ ^]^ The optical detection of amphiphilic toxins inside living cells was demonstrated by using the aqueous/LC interfaces of LC droplets (Figure 7c).^[^ ^45a^ ^]^ To provide physical and chemical robustness, the LC droplets enclosed in semi‐permeable polymer capsules were prepared for internalization by cells.

All‐atom MD simulation was used for studying molecular‐level phenomena at the aqueous/LC interfaces.^[^ ^20^ ^]^ MD simulations of thin films of typical nematic liquid crystals confined between vacuum and solutions of sodium iodide and sodium chloride showed the molecular reorientation of LC molecules at aqueous/LC interfaces by adding the ions.^[^ ^46^ ^]^ The free energy analysis for the transfer of a single water molecule from the bulk water phase to the LC phase was also discussed. Another MD simulation study demonstrated the organization of the nematic and isotropic phase of confining LC films between vacuum and the aqueous medium^[^ ^20b^ ^]^ and the monolayer of specific amphiphiles at the aqueous/LC interface caused the local configurational transitions of the underlying liquid crystals.^[^ ^20c^ ^]^

For understanding the detail mechanism of the protein adsorption or binding at aqueous interfaces, MD simulations have been powerful tool to investigate the dynamics of the interfacial molecules, biomolecules, and water molecules. The adsorption of the peptide on the solid surfaces,^[^ ^47^ ^]^ and the adsorption of the protein on graphite, self‐assembled monolayers,^[^ ^48^ ^]^ and the crystalline polyethylene surface^[^ ^49^ ^]^ were investigated by MD simulations. In these studies, the adsorption process and the conformation changes of biomolecules, the interaction energies between the biomolecules and the surface, and the effects of the surface treatment were quantitatively discussed.

**Figure 8:**
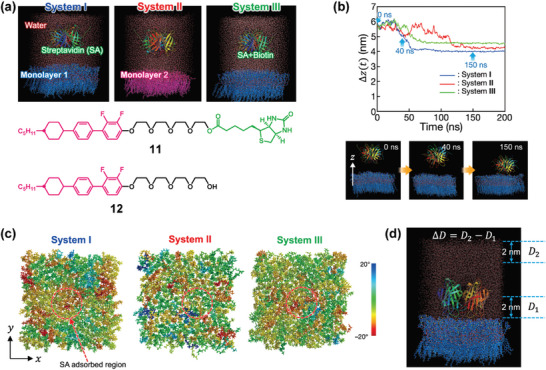
a) Representative snapshot from MD simulation for **Systems I**, **II**, and **III**. Monolayers 1 and 2 are composed of bioconjugated amphiphilic mesogen **11** and biotin‐free mesogen **12**, respectively. b) Time profiles of the relative distance along the layer normal direction normal to the monolayer between the center of the mass of streptavidin and the monolayer and snapshots of MD simulation for **System I** at 0, 40, and 150 ns. c) Distribution maps of the tilt angle difference of individual mesogens viewed from the top. d) *D*_1_ and *D*_2_ denote the diffusion coefficients of the water molecules moving in the domain within 2 nm from the water–monolayer and vacuum–water interfaces, respectively. Adapted with permission.^[^ ^21b^ ^]^ Copyright 2020, American Chemical Society.

While MD simulation has been well used for the study on the phenomena in which the biomolecules were adsorbed at the solid, solid‐like, or hydrophobic oil surfaces, the microscopic dynamics at the aqueous/LC interfaces have rarely been investigated. A recent MD simulation study have advanced our understanding of the molecular‐level phenomena involved in the adsorption of biomolecules and subsequent dynamic changes at the aqueous/LC interfaces (**Figure** **8**).^[^ ^21b^ ^]^ The monolayer composed of bioconjugated amphiphilic mesogen **11** ^[^ ^21^, ^41^ ^]^ containing biotin moiety significantly showed stronger adsorption with streptavidin than that of biotin‐free mesogen **12**.^[^ ^21^, ^41^ ^]^ During the process of the adsorption of streptavidin to the monolayer, the structure of streptavidin was almost maintained. The structural rigidity of streptavidin was increased with ligand‐binding. The dynamic behavior of LC molecules was significantly changed by the introduction of the biotin moiety into the mesogenic molecules: the streptavidin adsorption slightly increased the orientational orders.^[^ ^21b^ ^]^ The tilt angles for LC molecule **11** that bound stably to streptavidin decreased by more than ≈10°. The analysis of the diffusion behaviors of the water molecules in the vicinity of the aqueous/LC interface and in the bulk indicated that the adsorption of streptavidin decreased the self‐diffusion coefficients of the water molecules at the interface.^[^ ^21b^ ^]^ The stronger the streptavidin was adsorbed at the monolayer, the greater water diffusion was suppressed at the aqueous/LC interface. The calculation of the in‐plane self‐diffusion coefficients of the water molecules parallel to the director of monolayer and its transverse direction indicated that the adsorption of streptavidin mainly affected the diffusion along the director. Recently, all‐atom MD simulations were performed to study the behavior of free polymer surfactants between typical nematic liquid crystals and bulk water.^[^ ^50^ ^]^ While the interaction energies between the polymer and LC molecules or water molecules were quantitatively calculated, the change of the orientational order of LC molecules and the dynamics of water molecules were not investigated. The analysis of the microscopic behavior of both LC molecules and surrounding water molecules by using all‐atom MD simulations is highly important to understand the mechanism of phenomena at the aqueous/LC interfaces. It would provide new insights to develop new aquatic functional LC materials.

## 4 Conclusion and Perspective

The history about the relationship between water and liquid crystals^[^ ^51^ ^]^ started with lyotropic liquid crystals of amphiphilic molecules^[^ ^52^ ^]^ and colloidal liquid crystals of viruses such as tobacco mosaic virus.^[^ ^53^ ^]^ These water‐based liquid crystals have long history of more than 100 years. Recently, self‐assembled water treatment LC membranes transporting water in the nanopores^[^ ^8^ ^]^ and aqueous/LC interfaces as chemical and biological sensors^[^ ^9^ ^]^ have been developed. In these materials, key common issues are the interactions of water molecules and LC substances and the dynamics of water molecules.

Nanosegregated structures of micellar lyotropic liquid crystals exhibiting lamellar, hexagonal, and bicontinuous cubic structures have been known. For thermotropic liquid crystals, block‐structured molecules forming nanosegregated structures similar to those of lyotropic liquid crystals have attracted much attention.^[^ ^54^ ^]^ The molecular design and functionalization of these thermotropic LC materials have been intensively studied over the past two decades.^[^ ^3^, ^7^ ^]^ Further functionalization of the aquatic LC materials working with water such as water treatment LC membranes^[^ ^8^ ^]^ and aquatic LC sensors^[^ ^9^ ^]^ described in this review will be achieved by control of molecular alignment. To establish new molecular technology of liquid crystals related functional materials, combination of advanced measurements^[^ ^28^, ^55^ ^]^ and data sciences^[^ ^56^ ^]^ as well as simulations is important and essential. For example, new reentrant phase transition behavior of ion‐transporting smectic liquid crystals were found by experiments and explained by electron density profile analysis and large‐scale MD simulations.^[^ ^55e^ ^]^

Development of the aquatic functional liquid crystals as a class of functional soft materials interacting with water has made significant progress in recent years. The water treatment LC membranes exhibiting removal of viruses and harmful substances have been demonstrated.^[^ ^8^, ^23^ ^]^ Aquatic LC membranes have also potentials for gas separation because humid conditions are used for the membrane separation.^[^ ^57^ ^]^ Micellar lyotropic liquid crystals have been used as drug delivery carriers.^[^ ^58^ ^]^ New structures and properties of lyotropic LC molecular assemblies have also been explored to provide fundamental insights into development of functional materials as well as understanding of biological systems.^[^ ^59^ ^]^ Interactions of liquid crystals with cell membranes have been examined to obtain new biomaterials.^[^ ^60^ ^]^ Bioconjugated supramolecular LC polymer networks have potential as new stimuli‐responsive LC materials in aqueous environments.^[^ ^61^ ^]^ Design of biomimetic and hybrid LC materials is of interest as aquatic functional materials.^[^ ^62^ ^]^ The dynamic processes of molecular assembly and the phase transitions of the aquatic LC materials are difficult to observe experimentally and they remain to be elucidated; however, the recent development of MD simulations allows us to capture such microscopic dynamics at molecular level in bulk materials.^[^ ^55a,b^ ^]^ Data science also helps the new design of aquatic materials for a variety of functions.^[^ ^56^ ^]^ Since water exists in living organisms^[^ ^63^ ^]^ and on the earth, aquatic functional liquid crystals will be more important for applications in the fields of environment, resources, medical care, and biotechnology for our sustainable development.

## Conflict of Interest

The authors declare no conflict of interest.
